# How to Optimize the Results of Liver Transplantation for Acute-on-Chronic Liver Failure

**DOI:** 10.3390/life13051152

**Published:** 2023-05-10

**Authors:** Sophie-Caroline Sacleux, Faouzi Saliba

**Affiliations:** Hepato-Biliary Center, AP-HP Paul Brousse Hospital, Paris-Saclay University, INSERM Unit N°1193, 94800 Villejuif, France; sophie-caroline.sacleux@aphp.fr

**Keywords:** acute-on-chronic liver failure, liver transplantation, cirrhosis, intensive care unit

## Abstract

Cirrhotic patients who developed a decompensation episode requiring an admission to an intensive care unit are not equal in term of prognosis. This led to the definition of a syndrome, acute-on-chronic liver failure (ACLF), marked by the severity of systemic inflammation, the development of organ failures and a high short-term mortality. The most common underlying liver etiology is related to acute alcohol hepatitis in western countries and to HBV or HCV cirrhosis in eastern countries. Twenty-eight and 90-days high mortality rates are well linked to the number of organ failure and defined, merely ten years ago, by a modified SOFA score. ACLF is a dynamic syndrome and grading can vary from hospital admission. ACLF grading between day 3–7 of admission is more accurate for determining outcome. ACLF-3 patients with ≥3 organ failures remain very challenging with >75% mortality rate. Despite recent advances in the medical management of critically ill cirrhotic patients, the prognosis of these patients remains poor. Currently, the main effective treatment is an urgent liver transplantation (LT) which is performed in a very selected patients eligible to transplant given the limited availability of organ donors and the low post-transplant survival rates reported in earlier studies. Recently, large retrospective multicenter studies and registries showed an improved 1-year post-transplant survival rate >83% in several transplant centers. Nevertheless, only few proportions of the ACLF-2 and ACLF-3 patients are transplanted representing 0–10% of most liver transplant programs. A careful selection of these patients (excluding major comorbidities i.e., older age, addictology criteria, severe malnutrition…) and optimal timing for transplant (infection control, hemodynamic stability, low oxygen and vasopressor requirements) are associated with excellent post-transplant survival rate.

## 1. Introduction

The prognosis of cirrhotic patients admitted to intensive care units for a decompensating episode has improved since the 2000s, with an estimated decreased in mortality of about 15%. Nevertheless, those patients with multi-failure organs (i.e., at least 3 organ failures) still have a very poor prognosis [1]. This improvement is due to the description and understanding of Acute-on-Chronic Liver Failure (ACLF) but also to the increasing access of these cirrhotic patients to emergency liver transplantation from intensive care units.

Liver transplantation in ACLF has been reported in retrospective series and registries with contrasting results. Post-liver transplantation survival rates for ACLF-3 varied from 52 to 93% at 1 year [2]. These differences are probably related to differences in patient selection and centers experience in critically ill patients. The present narrative review will provide insights and updates on the current understanding of ACLF with emphasis on selection of patients to transplant, general and specific management of patients prior to transplant and timing for liver transplantation, to optimize post-transplant results.

## 2. Definition and Grading of ACLF

Acute-on-Chronic Liver Failure is a severe form of acute decompensation of cirrhosis, characterized by systemic inflammation, organ failures and a poor short-term prognosis. In 2013, the CANONIC study conducted by the European Foundation for the study of Chronic Liver Failure (EF-CLIF) collected data prospectively from 1343 decompensated cirrhotic patients in 29 European hepatology centers [3]. This study defined chronic liver failure (CLIF), associated organ failure and grades associated with 28- and 90-day mortality. A CLIF-C OF score for CLIF Consortium Organ Failure (adapted from the SOFA score used to define organ failure and prognosis in patients with sepsis), was constructed to define organ failure and ACLF grades (Table 1) [4]. Based on the number and type of failures, ACLF grades from 1 to 3 are defined. ACLF grade 1 was defined by either a single kidney failure or a non-kidney organ failure if associated with kidney impairment or hepatic encephalopathy, while ACLF grade 2 corresponds to 2 organ failures and ACLF 3 to at least 3 organ failures [4]. Short-term mortality in ACLF patients increases with the number of failures: from 15% at 28 days in patients with one organ failure to 80% if there are more than 3 organ failures [5]. Similarly, ACLF grade is linearly associated with mortality with ACLF 1 patients achieving >23% mortality at D28, ACLF 2 patients 31% and ACLF 3 superior to 75% mortality. The sensitivity and specificity of the score, as determined by the area under the ROC curve, are superior to the Model for End-Stage Liver Disease (MELD) and MELD-Na scores [4]. 

There are some differences in the ACLF definition between the European Association for the Study of the Liver-Chronic Liver failure (EASL-CLIF) defined above, the North American for the Study of End-Stage Liver Disease and the Asian Pacific Association for the Study of the Liver [6]. Definition variations are mostly related either to the underlying etiology, the presence or not of previous decompensating episode, the definition of organ failures, or to the presence or absence of extrahepatic organ failures. The most common precipitants are acute alcoholic hepatitis and bacterial infections in western countries and hepatitis viral B reactivation or flare in eastern countries. Despite that there are no comparative studies, mortality rates are proportional to the number of organ failures within the three definitions worldwide used. 

## 3. Pathophysiology of ACLF

A large progress has been made in the understanding of pathophysiological mechanisms of ACLF, with a great link between the development of inflammatory biomarkers, damage-associated molecular patterns (DAMPs) and pathogen-associated molecular patterns (PAMPs), bacterial translocation, immune paralysis and overall, the intensity of systemic inflammation and the number of organ failure. Acute alcoholic hepatitis (AAH), bacterial infection are main precipitating factors of systemic inflammation. In the context of AAH, systemic inflammation result from the release, by damaged and necrotic liver cells, of damage-associated molecular patterns (DAMPs) and in case of infection of pathogen-associated molecular patterns (PAMPs), both leading to an immune mediated tissue damage and single or multiple organ failure [6]. Claria et al. [7] investigated various inflammatory biomarkers in 522 patients with decompensated cirrhosis (237 patients with ACLF) and 40 healthy subjects. Systemic inflammation was assessed by measuring 29 well known cytokines and the redox state of circulating albumin (HNA2). Patients with ACLF showed significantly higher levels of inflammatory cytokines and biomarkers of circulatory dysfunction (i.e., copeptin and plasma renin) than those without ACLF. The severity of systemic inflammation and of ACLF at enrollment were strongly associated. The authors supported the main role of systemic inflammation as a primary driver in ACLF [7]. 

## 4. General Management of ACLF

In the European multicenter Canonic study, that lead to the definition and scoring of ACLF, 22% of the cirrhotic patients had ACLF at admission and merely 8% developed ACLF during hospital stay [3]. ACLF-2 and 3 patients would require in most of the cases an ICU admission for organ support whether the patient is listed for transplant or not. The eligibility for transplant requires a multidisciplinary team discussion, and often taken within few days of admission. Serial ICU and liver scores measurements during hospital stay are of major help in determining outcome and futility.

An early diagnosis and management of ACLF in specialized liver unit is mandatory to prevent irreversible organ damage. There is currently no identified therapeutic target [5], and management is most often limited to treatment of the precipitating event and organ support. Recently, Bernal and colleagues developed various practical considerations for the critical care management of these patients [8]. In summary, these consists on the following: (1) identification and treatment of precipitating event (i.e., infection, acute alcoholic hepatitis, drug toxicity…); (2) imaging of liver and hepatic vasculature; (3) microbiological screening and cultures; (4) for circulatory systemic support, a rapid assessment of fluid responsiveness and the use of norepinephrine as primary vasopressor, to maintain an adequate mean aortic pressure; terlipressin could be used as an adjunctive drug; (5) for renal support, early intervention to prevent and treat acute kidney injury (AKI), continuous modes of renal replacement therapy (RRT) are preferred to intermittent RRT; (6) identify and treatment of causes of reduced consciousness and minimal use of sedation (7) metabolic and nutritional support; (8) a close monitoring for nosocomial infection with routine and guided cultures at admission and during ICU stay; previous hospitalizations, infections and colonization needs special attention as well as those patients with severe malnutrition and under corticosteroids because of increased risk of sepsis induced mortality; (9) appropriate antibiotic use guided on patient cultures and the ecology of the department. 

## 5. Specific Management of ACLF

Artificial liver support devices, using the molecular adsorbent recirculating system (MARS™, Baxter International Inc., Deerfield, IL, USA), or the fractionated plasma separation and adsorption system Prometheus™ (Fresenius Medical Care, Bad Homburg, Germany), have failed in randomized controlled trial to improve survival in patients with ACLF. More recently, when considering the ACLF grading, albumin dialysis with MARS™ has shown to improve short-term survival (14 days), the efficacy appears to be correlated with patient selection and intensity of treatment [9]. Plasma exchange has shown to improve survival in ALF and has been used in eastern countries in non-randomized trials in ACLF patients. A multicenter international randomized controlled trial is currently ongoing to evaluate the efficacy and safety of Plasma exchange in patients with ACLF (NCT03702920). Meanwhile, the recommendations from the international expert panel recommended the use of extracorporeal albumin devices in patients listed for a transplant or with a transplant project or in clinical trials in specialized centers [9]. 

## 6. Liver Transplantation in ACLF

Despite recent advances in the medical management of advanced liver disease, liver transplantation (LT) remains the only intervention improving the prognosis of ACLF patients, in order to stop intrahepatic inflammation responsible of organ failure via systemic inflammation and to restore immunity [5]. These patients should preferably be referred rapidly to transplant centers. In France, liver transplantation for ACLF-3 concerns about 5% of transplants. In the European multicenter study by Belli et al. from 20 LT centers and 8 European countries, France and Germany reported the highest rate of LT for ACLF-2 and 3, respectively 27% and 41% of the 308 patients listed for transplant [10]. In a study published in 2022 by Artzner et al., ACLF patients represented 14% of patients transplanted for decompensated cirrhosis in France, with significant variations depending on the transplant center, from 6.6 to 22.8% [11].

**Figure 1:**
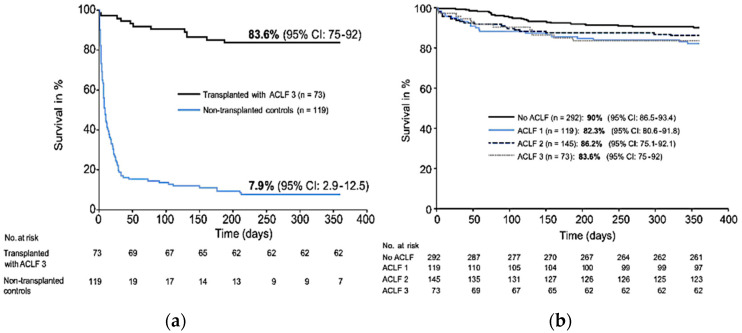
(**a**) 1-year survival of patients transplanted with ACLF-3 and non-transplanted matched controls with cirrhosis and multiple organ dysfunction. (**b**) 1-year survival of transplanted patients according to the ACLF grade. Adapted with permission from Ref. [12].

In the literature, liver transplantation in ACLF has been studied in retrospective series with contrasting results. Indeed, post liver transplantation survival rates for ACLF-3 vary from 52 to 93% at 1 year [2,10,11,12,13,14,15]. Among the largest series published in the literature, the French multicenter study of Artru et al. involving 73 patients transplanted with ACLF-3 showed interesting results [12]. First, there was a significant difference in 1-year survival in ACLF-3 transplanted patients (83.6%) compared to non-transplanted ACLF-3 patients (7.9%; *p* < 0.0001) (Figure 1a). In addition, there was no significant difference in 1-year post-transplant survival compared to ACLF-1, 2 or non-ACLF transplant patients (Figure 1b). In contrast, there was a higher length of stay in ACLF-3 transplant patients and a significantly higher postoperative complication rate. More nuanced results have been found in other retrospective series, with notably the Strasbourg team in 2017 reporting a 1-year survival of 60% (n = 55) [13]. Apart from these French experiences, European and North American data report post-transplant survival rates of ACLF-3 patients of 81–84% at 1 year [10,15]. Sundaram et al. reported retrospective data from the United Network for Organ Sharing (UNOS) registry of recipients listed from 2005 till 2016. Of the 100,594 patents identified on the waitlist, 6079 (6.0%) had ACLF-2, and 5355 (5.3%) had ACLF-3; overall, 50,552 received liver transplants. Patients with ACLF-3 were more likely to die or be removed from the waitlist, compared to the other ACLF groups. After transplantation, the 1-year survival was lowest among patients with ACLF-3 (81.8%) compared to other patient groups with low grade or no ACLF (88.1–91.9%, *p* < 0.001) [15]. 

The lack of consensus regarding the selection of candidates for transplantation and the pre- and post-operative management of patients might explain these disparities in post-transplant survival. Interestingly, we note an evolution of the post-transplant prognosis of ACLF-3 patients between 2007 and 2019 in the study of Michard et al. [16]. Indeed, survival improved significantly from 66% to 86% (*p* = 0.02). A better preoperative management and a better selection of patients and grafts were probably at the origin of this improvement. 

The main cause of post-transplant death in these critically ill patients are related to sepsis mainly due to systemic, pulmonary, surgical site or biliary infections with multidrug resistant bacteria (MDRB). Many of these patients has been colonized prior to transplant during their hospital stay. The risk of developing systemic infection or surgical site infection with the same microorganism is high with a large impact on mortality. Post-transplant invasive fungal infection is another major risk of mortality occurring mainly in high-risk patients as those with a MELD score >30 or with acute liver failure [17]. Fungal infections either due to *Candida* species with low susceptibility or resistant to azoles (i.e., *Candida glabrata*, *Candida Krusei*) are common in the liver transplant recipients. Invasive aspergillosis commonly occurs in patients with ACLF who received corticosteroids for the treatment or acute alcoholic hepatitis. Screening for these patients at time of transplant is mandatory. Fungal prophylaxis for 2–4 weeks has been strongly recommended by the International Liver Transplant Society (ILTS) for high-risk patients and these includes patients with a MELD-score >30 and ACLF patients [18]. 

## 7. Selection of Liver Transplantation Candidates

Selection, although appearing to be a key element of success, remains a subject of debate for transplantation teams: there is currently no consensus, either on the criteria for patient selection or on the ideal time for transplantation. Given the impact of this procedure on the prognosis, it is essential to discuss a liver transplantation project for each cirrhotic patient entering the ICU. 

ACLF is a dynamic syndrome as patients might develop a rapid fatal outcome within few days of admission and others might improve their ACLF grading allowing opportunities to compensation or transplantation. ACLF grading between day 3 to 7 is better predictive of outcome compared to grading at ICU admission. The first step of the selection process consists of a rapid evaluation of the patient upon admission to the ICU to eliminate absolute contraindications to LT. The minimal assessment will therefore include the search for serious comorbidities (i.e., cardiovascular, pulmonary), severe malnutrition, active cancer and severe infection that could compromise the transplantation project in the immediate future. Age has been shown that impact outcome of ACLF and therefore determine the eligibility to transplant and is discussed below.

Infection leading to sepsis, severe sepsis or septic shock is common in these patients. A 48–72-h adequate infection control and hemodynamic stability are essential. Cultures from various sites for detecting multidrug resistant organism and screening for fungal infection by biomarkers and imaging (i.e., invasive aspergillosis, endocarditis...) are mandatory. As excessive alcohol consumption is observed in 80% of ACLF patients in Europe, an addictology and psychosocial evaluation inspired by the Mathurin et al. study for emergency liver transplantation of patients with acute alcoholic hepatitis is also essential [19]. Several studies have shown that relapse following LT for AH ranges between 10 and 25% within the first 2–3 years [20,21,22]. In a recent prospective study, alcohol relapse was detected in 34% at 2 years compared to 25% in those transplanted for alcohol-associated cirrhosis with 6-month abstinence pre-LT [23]. Despite a drastic selection of alcoholic cirrhotic patients, a relapse rate was still observed in merely 25% of the patients without affecting 5-year patient and graft survival [20,21,22,23]. 

**Figure 2:**
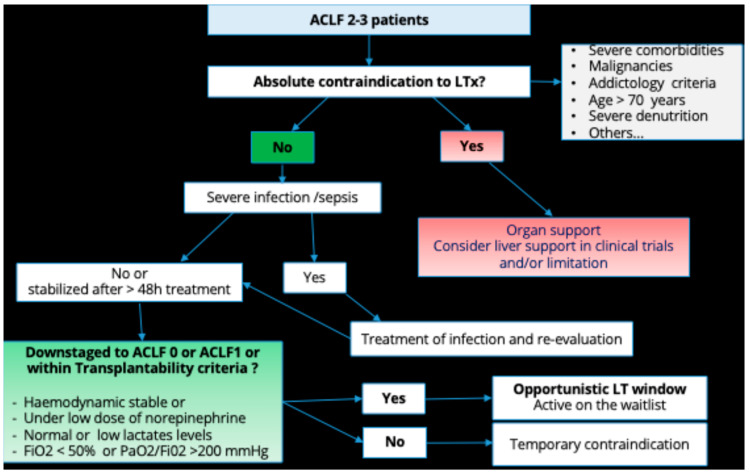
Decisional tree for selection of cirrhotic patients with ACLF admitted to ICU to transplant.

At the end of this assessment, in the setting of our prospective study, approximately one patient out of two could be a candidate for liver transplantation [24]. The main causes of non-eligibility to transplant are mostly related to age, severe comorbidities, and absence of abstinence. Non-alcohol abstinence and therefore a high expected alcohol relapse is the main frame to transplant in a context of paucity of organ donors. A decisional tree for selection of cirrhotic patients with ACLF admitted to ICU to transplant is illustrated in Figure 2.

## 8. Timing of Liver Transplantation

Once the evaluation has been carried out and the decision for liver transplantation (LT) has been made, the next and most important step is to choose the best time to transplant the patient. Currently, patients with ACLF are not given priority on the national transplant list, as is the case for those patients listed for ALF. Indeed, due to the presence of organ failure in very fragile patients, transplantation in ACLF patients is considered at high risk of post-operative morbidity and mortality.

The natural history of ACLF alternates between phases of worsening and phases of stabilization during which the patient could be transplanted. There is often a short period, a transplant window, to perform the transplant in an optimal condition. In 2020, Sundaram et al. showed that an improvement in the number of organ failures and the ACLF grade between listing and transplantation was associated with a better prognosis [25]. The challenge in making the decision to transplant is therefore to wait for improvement without waiting for the next deterioration or even terminal liver failure. 

Numerous retrospective studies have looked at the risk factors for postoperative mortality. Among them, mechanical ventilation is a very clearly identified factor in all the studies and a fortiori the PaO2/FiO2 ratio, reflecting the patient’s oxygenation [10,13,24]. On the other hand, the results on the other parameters of “extra-hepatic” organ failure (extra-renal purification, use of catecholamines) remains unclear [10,24]. These parameters probably need to be evaluated more finely: catecholamine doses, cause of extra-renal purification, among others. Lactates also seem to play a role with a threshold found at 4 mmol/L in several studies [10,13]. In 2021, Weiss et al. reported the results of a national survey on the criteria to transplantation of ACLF patients [26]. Despite heterogeneous results, the following criteria were proposed to contraindicate transplantation: norepinephrine dosage >1 µg/kg/min, a PaO2/FiO2 ratio <150 mmHg and arterial lactates >9 mmol/L. This study also highlights the variability of the criteria used from one team to another, and even from one practitioner to another, and the complexity of the decision making. In this context, the transplantation for ACLF-3 model (TAM score) published by Artzner et al. in 2021, which includes age, arterial lactates, PaO2/FiO2 ratio, and leukocyte count (G/L), may be useful in deciding the timing of transplantation [27]. The TAM score was derived by assigning 1 point to each of the variables affecting outcome. A cut-off at 2 points distinguished a high-risk group (score > 2) from a low-risk group (score ≤ 2) with 1-year survival of 8.3% vs. 83.9% respectively (*p* < 0.001) [27]. We recently evaluated retrospectively the TAM score in our previously reported cohort of 73 ACLF patients. As in the initial publication, the score was calculated at time of transplantation. The survival was respectively 83.3% (95%CI:72.3–92.3) in patients with a TAM score ≤ 2 vs. 80.0% (95%CI: 44.9–100), *p* = 0.89 in those with a TAM score > 2 [28]. In our series, only 7% of the patients had a TAM score > 2 suggesting that the selection process had considered the variables included in the TAM score. 

In the study of Artru et al. from our group, we contraindicate to transplant those ACLF patients with active gastrointestinal bleeding, control of sepsis for less than 24 h, hemodynamic instability requiring noradrenaline >3 mg/h (equivalent to 0.6 µg/kg/min) and severe acute respiratory distress syndrome (PaO2/FiO2 ratio <150 mmHg). The median time between listing and LT was eight days and the delay between ICU admission and transplantation ranged from 7–11 days, confirming that these patients have a short transplantation window [12,29]. Thuluvath et al. has shown that if a liver transplant is performed quickly, the 1-year post-transplant survival rate is high, ranging from 84% with three organ failures to 81% with 5–6 organ failures [30,31]. Therefore, the decision for listing should be taken within a narrow period. 

Whether these patients should benefit from a prioritization score as for ALF, is still debated. Nevertheless, these patients have, in merely all the cases, a priority access as they have a high MELD or MELD-Na score; but the access to transplant vary among countries. 

Two additional factors have been identified and correlated with post-transplant outcome. Age with limitations for transplant to 50, 55 and 60 years according to studies [10,27]. The threshold of age associated with poorer outcome following LT was 60 years old in the studies from registries of ELTR and OPTN. Age should not probably be considered alone but in the setting of presence of other comorbidities (i.e., cardio-vascular, diabetes, sarcopenia…). Finally, several studies underline the importance of graft quality [15], notably by calculating the Donor Risk Index (DRI) as a factor of post-transplant survival. They demonstrated a greater 1-year survival when the recipient was transplanted with a low-risk organ (76.5%) vs. suboptimal organ (71.6%) (*p* = 0.034). 

## 9. Inequity of Access to Liver Transplantation in ACLF

All over the world, MELD score is a major criterion for the allocation of transplants on the national waiting list, prioritizing the most severe decompensated cirrhosis patients. The “sickest first” principle theoretically offers easy access to transplantation for ACLF patients because of their high MELD score. In the French National CRISTAL registry study, 61% of cirrhotic patients transplanted from the ICU had a MELD score >30 [11]. The waiting time on the transplant list for an ACLF-3 patient is relatively short, on average of 5 days in Europe, as reported by Belli et al. [10]. The high-emergency system used for listing ALF is therefore not used for transplantation of ACLF patients. Nevertheless, a reassuring post-transplant survival and an obvious individual benefit have led some authors to call for the implementation of a specific prioritization for ACLF patients [15]. At present, there is no consensus on this prioritization, mainly because of the lack of long-term follow-up of these patients, with few data on patient and graft survival beyond one year. Nevertheless, it is being tested in Spain and the United Kingdom. There is a great disparity in access to liver transplantation for ACLF patients, depending on the center, both in France and in Europe [10,11,32]. This underlines the fact that urgent transplantation of ACLF patients depends on the practices of the teams, mainly related to the question of collective benefit and the equity of this type of transplantation in the context of a shortage of grafts.

Although theoretically eligible for liver transplantation, ACLF patients are not always transplanted in clinical practice due to the severity of ACLF, with a high mortality rate on the transplant list. In the European ELITA/EF-CLIF registry, a list mortality of 24% was observed for all ACLF grades combined, with a 1-year survival of listed ACLF-3 patients of 53% [10]. In a single-center French study of 200 cirrhotic patients admitted to the intensive care unit of the Paul Brousse Hospital liver transplantation center, 28% of patients eligible for transplantation died before listing and the listed mortality rate was 26% [24]. We established a decisional tree that might help selecting ACLF 2–3 patients admitted to ICU for liver transplantation (Figure 2). 

## 10. Conclusions

Liver transplantation is currently the only treatment that can improve the very poor prognosis of cirrhotic patients in intensive care. The good survival data, close to that of a patient not hospitalized in the ICU despite a high number of organ failures, have led many teams to perform transplantation. The current survival rate of patients transplanted for ACLF-3 rate improved with time and transplant team experience and currently reaching 85 to 90%. Nevertheless, the number of patients who could be transplanted in ACLF, the selection criteria of the candidates and the timing of the transplantation remain points to be elucidated. Major critical situations that require an early diagnosis and management prior to transplant, in patients with ACLF-3, to optimize liver transplantation results, are severe septic shock with hemodynamic instability and lactic acidosis, acute respiratory distress syndrome and fungal infection. Managing these situations, downstaging ACLF grading prior to transplant, and avoiding “futility” is associated with better post-transplant survival.

## 11. Future Perspectives

− There are still inequalities in access to intensive care services and a fortiori to liver transplantation for these patients depending on the centers.
− Liver transplantation in ACLF will continue to develop in the coming years, in parallel with the evolution of resuscitation techniques, as well as those of organ retrieval and conservation and this would further improve the results.
− Increased collaboration and communication between the resuscitation and transplantation teams appears to be a key element in the progression of this technique, to ensure more equitable access to care for ACLF patients.
− New scoring system evaluating more accurately the decision and timing of transplant would need further evaluation and validation in prospective cohorts.
− The use of machine perfusion to improve the quality of the graft and extend the pool of graft, in addition to improvement in technical surgical skills would improve the results.
− An international EF-CLIF study (CHANCE, liver transplantation in patients with CirrHosis and severe ACLF: iNdications and outcome) is currently underway to try to answer these questions and improve the pre- and post-operative management of these patients. The study will collect numerous biological samples to explore biomarkers and pathophysiological mechanisms of the disease, including organ recovery.
− Artificial liver support using new devices (The DIALIVE^®^ system), high and low volume plasma exchange, hemadsorption might have an essential role following results of ongoing trials either to improve transplant-free survival or downstaging ACLF grade.
− The development of mesenchymal liver Human allogeneic liver-derived progenitor cells (HepaStem^®^; Promethera Biosciences, Mont-Saint-Guiber, Belgium; Cellaïon^®^) targeting the inflammatory process to improve liver regeneration and function in patients with ACLF, currently in trials, might be promising [33].

## Figures and Tables

**Table 1:** The CLIF-organ failure score system. In red, criteria for diagnosing organ failures. * Brain, West-Haven grade for hepatic encephalopathy; FiO2, fraction of inspired oxygen; PaO2, partial pressure of arterial oxygen; SpO2, pulse oximetric saturation. Adapted with permission from Ref. [4].

Organ/System	Subscore 1	Subscore 2	Subscore 3
Liver	Bilirubin < 6 mg/dL	Bilirubin ≥ 6 mg/dL and <12 mg/dL	Bilirubin ≥ 12 mg/dL
Kidney	Creatinine < 2 mg/dL	Creatinine ≥ 2 mg/dL and <3.5 mg/dL	Creatinine ≥ 3.5 mg/dL or renal replacement
Brain *	Grade 0	Grade 1–2	Grade 3–4
Coagulation	INR < 2.0	INR ≥ 2.0 and <2.5	INR ≥ 2.5
Circulation	MAP ≥ 70 mmHg	MAP < 70 mmHg	Use of vasopressors
Respiratory			
PaO2/FiO2	300	≤300 and >200	≤200
or	or	or	or
SpO2/FiO2	>357	>214 and ≤357	≤214

## Data Availability

Not applicable.
